# Transgender women on oral HIV pre‐exposure prophylaxis have significantly lower tenofovir and emtricitabine concentrations when also taking oestrogen when compared to cisgender men

**DOI:** 10.1002/jia2.25405

**Published:** 2019-11-06

**Authors:** Eugenie Shieh, Mark A Marzinke, Edward J Fuchs, Allyson Hamlin, Rahul Bakshi, Wutyi Aung, Jennifer Breakey, Tonia Poteat, Todd Brown, Namandjé N Bumpus, Craig W Hendrix

**Affiliations:** ^1^ Department of Medicine (Clinical Pharmacology) Johns Hopkins University School of Medicine Baltimore MD USA; ^2^ Department of Pathology Johns Hopkins University School of Medicine Baltimore MD USA; ^3^ Department of Social Medicine University of North Carolina Chapel Hill Chapel Hill North Carolina USA; ^4^ Department of Medicine (Endocrinology) Johns Hopkins University School of Medicine Baltimore MD USA

**Keywords:** HIV pre‐exposure prophylaxis, drug‐drug interaction, transgender women, tenofovir, emtricitabine, gender‐affirming hormonal treatment

## Abstract

**Introduction:**

Oral HIV Pre‐Exposure Prophylaxis (PrEP) with tenofovir (TFV) disoproxil fumarate (TDF)/emtricitabine (FTC) is highly effective. Transgender women (TGW) have increased HIV risk, but have been underrepresented in trials. For TGW on oestrogens for gender‐affirming hormone treatment (GAHT), TDF/FTC‐oestrogen interactions may negatively affect HIV prevention or gender‐affirming goals. Our aim was to evaluate any pharmacokinetic drug‐drug interaction between GAHT and TDF/FTC.

**Methods:**

We performed a pharmacokinetic study, in an urban outpatient setting in 2016 to 2018, of the effects of GAHT on TFV, FTC and the active forms TFV diphosphate (TFV‐DP) and FTC triphosphate (FTC‐TP) in eight TGW and eight cisgender men (CGM). At screening, participants were HIV negative. TGW were to maintain their GAHT regimens and have plasma oestradiol concentrations >100 pg/mL. Under direct observation, participants took oral TDF/FTC daily for seven days. At the last dose, blood was collected pre‐dose, one, two, four, six, eight and twenty‐four hours, and colon biopsies were collected at 24 hours to measure drug concentration. TGW versus CGM concentration comparisons used non‐parametric tests. Blood and colon tissue were also obtained to assess kinase expression.

**Results:**

Plasma TFV and FTC C_24_ (trough) concentrations in TGW were lower by 32% (*p* = 0.010) and 32% (*p* = 0.038) respectively, when compared to CGM. Plasma TFV and FTC 24‐hr area under the concentration‐time curve in TGW trended toward and was significantly lower by 27% (*p* = 0.065) and 24% (*p* = 0.028) respectively. Peak plasma TFV and FTC concentrations, as well as all other pharmacokinetic measures, were not statistically significant when comparing TGW to CGM. Oestradiol concentrations were not different comparing before and after TDF/FTC dosing. Plasma oestrogen concentration, renal function (estimated creatinine clearance and glomerular filtration rate), and TFV and FTC plasma concentrations (trough and area under the concentration‐time curve) were all correlated.

**Conclusions:**

GAHT modestly reduces both TFV and FTC plasma concentrations. In TGW taking GAHT, it is unknown if this reduction will impact the HIV protective efficacy of a daily PrEP regimen. However, the combination of an on demand (2 + 1 + 1) PrEP regimen and GAHT may result in concentrations too low for reliable prevention of HIV infection.

## 1 Introduction

Transgender women (TGW) are at high risk for HIV transmission with a worldwide prevalence of 19% 1, making them a critical population for HIV prevention with pre‐exposure prophylaxis (PrEP). While PrEP willingness in TGW is high 2, 3, several challenges limit PrEP use and efficacy. Barriers to PrEP uptake in TGW include concerns about side effects and the effect of PrEP drugs on gender‐affirming hormone treatment (GAHT), limited transgender‐inclusive PrEP promotion, and medical mistrust 4. The iPrEx trial of oral daily tenofovir disoproxil fumarate (TDF) 300 mg/emtricitabine (FTC) 200 mg (Truvada^TM^ Gilead Sciences, Inc. Foster City, CA) showed efficacy in men who have sex with men (MSM) and TGW, collectively 5. However, a subgroup analysis of TGW on GAHT showed lower PrEP efficacy and lower adherence (as measured by pharmacologic assessment) when compared to MSM and TGW not on GAHT; also, the pharmacologically based adherence benchmarks used were established in persons not on GAHT 6. Most TGW use GAHT, including oestrogen, to induce feminization 7. In one survey, 86.6% of TGW cited unwillingness to use PrEP due to worries that their hormone regimens may be affected 8. Surgical interventions for both hormonal modification (e.g. orchiectomy) and anatomic modification ranging from silicone injections to gender reassignment surgery are also elected, though with less frequency.

The impact of GAHT on TDF/FTC metabolism is poorly understood. The activation of tenofovir (TFV) and emtricitabine (FTC) requires intracellular phosphorylation by different nucleotide kinases in different tissues. In cell culture, oestrogen increases the activity of creatine kinase, which phosphorylates TFV‐MP in colon cells 9. Cisgender female genital tract CD4 + cells shows decreased TFV‐diphosphate (TFV‐DP) concentrations in the presence of progesterone and oestradiol 10. Ex vivo, oestrogen increases cytosolic 5’‐nucleotidase activity in only some human cervicovaginal cell types, but there are no data in colorectal cells 10. In vitro, the uptake of TFV and FTC into cervicovaginal cell lines increases or decreases varying with timing of oestrogen addition and cell type 11. Clinically, pregnancy is associated with high levels of endogenous oestrogen compared to the non‐pregnant state and changes in renal physiology significantly increase the clearance of many renally cleared drugs. The complex relationship between hormones and TDF/FTC uptake, activation and clearance make it challenging to predict the pharmacokinetic (PK) interaction between GAHT and TDF/FTC.

The aim of the study was to investigate whether drug in blood plasma, peripheral blood mononuclear cells (PBMCs) and colon tissue, following daily oral TDF/FTC dosing differed in TGW on GAHT when compared to cisgender men (CGM).

## 2 Methods

### 2.1 Objectives

This was a phase one open‐label PK interaction study of oral TDF/FTC and GAHT in TGW and CGM. The study was conducted in an urban outpatient setting in Baltimore, Maryland, from April 2016 to April 2018, after approval by the Johns Hopkins Medicine Institutional Review Board. The primary objective was to compare steady‐state plasma, intracellular PBMC, colon tissue homogenate, and colon tissue cell TFV, TFV‐DP, FTC and FTC‐TP concentrations in TGW on oestrogen and CGM not on oestrogen. Other objectives included assessing the effect of exogenous oestrogen on both nucleotide kinase expression of enzymes relevant for intracellular phosphorylation of TFV and FTC and colon tissue HIV infectivity assessed by ex vivo HIV challenge of colon tissue explants.

### 2.2 Study participants

Eligible participants were healthy HIV‐seronegative self‐identified TGW and CGM aged 18 to 65 years recruited from Baltimore, Maryland. TGW had to be taking oestrogen and have a serum total oestradiol concentration >100 pg/mL indicating consistent oestradiol use. The oestrogen formulation, dose, route and frequency were not otherwise restricted; other GAHT medications could be taken. Exclusion criteria included enrolment in HIV clinical trials, colorectal symptoms, rectal infection, hepatitis B surface antigen (HBsAg), altered gastrointestinal anatomy and concomitant medications with potential for toxicity or interactions.

### 2.3 Study procedures

After informed consent, participants were screened with history, physical, and laboratory evaluation for haematology, chemistries, HBsAg, HIV and syphilis; TGW had serum oestradiol measurements. Eligible participants underwent daily directly observed dosing of oral TDF/FTC in the research clinic each morning from Day 0 to Day 7 to assure TFV‐DP and FTC‐TP reached steady state 12, 13. Due to varied GAHT regimens, the only GAHT dose managed by the study team was on Day 7 when they were held until after pre‐dose (C_0_) blood collection. Plasma was sampled for TFV and FTC on Day 7 pre‐dose, one, two, four, six, eight and twenty‐four hours (C_24_) post‐dose. PBMCs were collected pre‐dose, two, eight and twenty‐four hours post‐dose for TFV‐DP, FTC‐TP and kinase expression. Serum hormones were collected 24 hours after the final TDF/FTC dose (Day 7, C_24_). Flexible sigmoidoscopy with 30 rectosigmoid biopsies was performed approximately 24 to 26 hours after the final TDF/FTC dose for histology, PK, kinase expression and ex vivo explant challenge.

### 2.4 Histology

Histology was pathologist scored for surface denudation (zero to three scale indicating thirds of epithelial surface affected), lamina propria haemorrhage (zero to three scale indicating thirds of lamina propria affected) and number of apoptotic bodies per 20× field as described previously 14.

### 2.5 Pharmacokinetic sample processing

For blood processing in all participants, plasma was prepared by centrifugation of coagulated blood in serum separator tubes at 1500 × g for 10 minutes at 4°C, aliquoted into cryovials, and stored at −80°C until analysis. PBMCs were isolated via centrifugation of a cell preparation tube (CPT) at 1800×g for 20 minutes at 20°C to 25°C, washed once with phosphate‐buffered saline (PBS), and centrifuged at 400 × g for 15 minutes at 4°C. Cells were resuspended in 10 mL PBS for cell counting. Cells were centrifuged again at 400 × g for 15 minutes at 4°C. Cell pellets were lysed with 2 mL of 70% ice‐cold methanol in water and stored at −80°C until analysis.

Colon biopsies were collected via flexible sigmoidoscopy 10 to 20 cm from the anus using 3.7‐mm pinch biopsy forceps (Microvasive no. 1599; Boston Scientific Corp., Natick, MA). Biopsies were placed in RPMI medium with l‐glutamine and 10% foetal bovine serum (R10 media) until processing. Biopsies for drug concentration were weighed and stored −80˚C prior to analysis. To release colon tissue cells for intracellular drug analysis, biopsies were incubated with an enzyme cocktail (collagenase type II, DNase I) in RPMI containing l‐glutamine, HEPES and 10% foetal bovine serum (FBS) using the 37C_h_TDK3 tumour dissociation programme on a GentleMACS (Miltenyi) tissue dissociator 13. Cells were counted via the Guava/Millipore EasyCyte Plus (Millipore, Billerica, MA). Thereafter, cells were processed similarly to the PBMCs.

### 2.6 Hormone sample processing and analysis

Serum for hormone analysis was per protocol of Brigham Research Assay Core (BRAC; Brigham and Women’s hospital, Boston, MA), which performed the assays, including LC‐MS for total oestradiol and total testosterone, tracer equilibrium dialysis for free testosterone, and immunoassay for LH and FSH.

### 2.7 Drug concentration analysis

TFV, FTC, TFV‐DP and FTC‐TP concentrations were determined by previously described liquid chromatography‐tandem mass spectrometry (UPLC‐MS/MS) methods validated for biological matrix by the Clinical Pharmacology Analytical Laboratory (CPAL) 15, 16, 17. CPAL participates in the National Institutes of Health‐supported Clinical Pharmacology Quality Assurance (CPQA) programme of assay method review and approval and periodic proficiency testing 18. Assays were validated based on the Food and Drug Administration Guidance for Industry, Bioanalytical Method Validation and met all acceptability criteria. Assay lower limits of quantification (LLOQ) were: plasma TFV and FTC, 0.31 ng/mL; tissue TFV, 0.05 ng/sample (median LLOQ based on biopsy weights, 0.002 ng/mg); tissue FTC, 0.25 ng/sample (median LLOQ based on biopsy weights, 0.010 ng/mg); PBMC and tissue TFV‐DP, 5 fmol/sample; PBMC and tissue FTC‐TP, 50 fmol/sample. Intracellular metabolite concentrations were normalized to cell counts and tissue weights and reported as fmol/10^6^ cells and fmol/mg respectively. Median LLOQ values for TFV‐DP and FTC‐TP concentrations in PBMCs, colonic tissue cells, and tissue homogenates were 0.446 fmol/10^6^, 0.721 fmol/10^6^, and 0.208 fmol/mg and 4.46 fmol/10^6^, 7.21 fmol/10^6^ and 2.08 fmol/mg respectively.

### 2.8 Kinase expression

Tissue samples and PBMCs were lysed using 1 mmol/L phenylmethylsulfonyl fluoride (PMSF) and protease/phosphatase inhibitor cocktail (Cell Signaling Technology). Lysate was centrifuged at 6000 × g for 10 minutes at 4°C and supernatant was collected for protein determination using a BCA protein assay kit (ThermoFisher). A 50 µg protein sample was resolved by SDS gel electrophoresis and transferred to a nitrocellulose membrane. Membranes were probed using primary antibodies for adenylate kinase 2 (AK2), creatine kinase muscle (CKM), pyruvate kinase liver and red blood cell (PKLR), pyruvate kinase muscle (PKM) (all kinase antibodies from Fisher Scientific), GAPDH (Cell Signaling) or β‐actin (Cell Signaling). Visualization was performed using SuperSignal West Dura or Femto ECL Substrate (ThermoFisher) and a BioRad Gel Doc XR (BioRad).

### 2.9 Ex vivo colon tissue explant HIV challenge

Four colon biopsies were incubated for 2 hours each with 10^3^ TCID_50_ per mL of HIV‐1, strain Ba‐L from Advanced Biotechnologies, Inc. (Columbia, MD) in a 24‐well plate after which they were placed in four individual wells on Surgifoam rafts (Ethicon Endo‐Surgery, Inc., Somerville, NJ) in culture media for 15 days. On days 4, 7, 10 and 14 all culture media were harvested and replaced (except final day 14) with fresh media. Harvested media were assayed for HIV‐1 p24 antigen using the Alliance HIV‐1 ELISA kit (PerkinElmer, Waltham, MA) according to manufacturer’s instructions. Cumulative p24 antigen produced in supernatant over the incubation period was divided by biopsy weight for weight‐adjusted cumulative p24 antigen. Median (of four) biopsy weight‐adjusted cumulative p24 produced over 14 days was the unit of analysis 19.

### 2.10 Pharmacokinetic and statistical analysis

PK parameters in plasma and PBMC (area under the concentration vs. time curve from zero to 24 hours [AUC_0‐24_], peak concentration [C_max_]_,_ trough concentration [C_24_], steady‐state total clearance [CL_ss_/F], and volume of distribution based on the terminal phase [Vz/F]) and colon tissue concentration 24‐hours post‐dose (C_24_) were estimated using non‐compartmental analysis (Pharsight WinNonlin v. 8.1, Certara, Inc., Cary, NC) and summarized using median and interquartile range (IQR). Differences between gender groups were compared using non‐parametric Wilcoxon rank sum test; correlations between variables were assessed using Spearman rank correlation test (IBM SPSS, v. 25.0. Armonk, NY). We referred to *p* < 0.05 as statistically significant and <0.1 as trending toward statistically significant. The sample size enabled detection of a 1.4 effect size with 80% power and 5% two‐sided alpha error.

## 3 Results

### 3.1 Demographics and Clinical characteristics

Eight TGW and eight CGM were enrolled. Three‐quarters of participants in both categories were of African ancestry. TGW trended towards a statistically significant 35% higher BMI than the CGM (*p* = 0.061; Table 1). TGW self‐reported GAHT regimens varied widely and GAHT use ranged in duration from one to twenty‐seven years (Table 2). Six (75%) TGW were taking spironolactone in combination with various oestrogen regimens. At screening, median (IQR) plasma oestradiol concentrations were 756 (187 to 1370) pg/dL. No participants had previously elected gender‐affirming surgical interventions.

**Table 1:** Demographic characteristics

	TGW Median (IQR)	CGM Median (IQR)	*TGW/CGM* *%*	*p* value
Age, years	29 (26, 41)	46 (28, 52)	63	0.195^a^
Weight, kg	98 (83, 123)	83 (71, 91)	118	0.130
BMI	31 (24, 36)	23 (21, 27)	133	0.061
Race, n (%)				
Asian ancestry	1 (12)	0 ( 0)		1.000^b^
African ancestry	6 (75)	6 (75)		
European ancestry	1 (12)	2 (25)		

IQR, interquartile range.

a Exact 2‐sided *p* value, Wilcoxon rank sum test, comparing TGW and CGM

b Fisher’s exact test.

**Table 2:** Self‐reported hormone regimens of transgender women

PID	Years on GAHT	Oestradiol (oral)	Oestradiol Valerate (IM)	Premarin	Spironolactone	Medroxyprogesterone
1010	1			1.25 mg q day		
1011	2	6 mg q day			200 mg q day	
1012	4			1.25 mg q day	50 mg q day	
1017	3		0.5 mg q 2 weeks		50 mg q day	
1018	1	6 mg q day	20 mg q 2 weeks		200 mg q day	
1019	27	2 mg q day	40 mg q 2 weeks	6.25 mg q day		
1020	9		20 mg q 2 weeks		100 mg q day	5 mg q day
1021	7		1.5 mg q 1 week		200 mg q day	

### 3.2 PrEP PK

**Figure 1:**
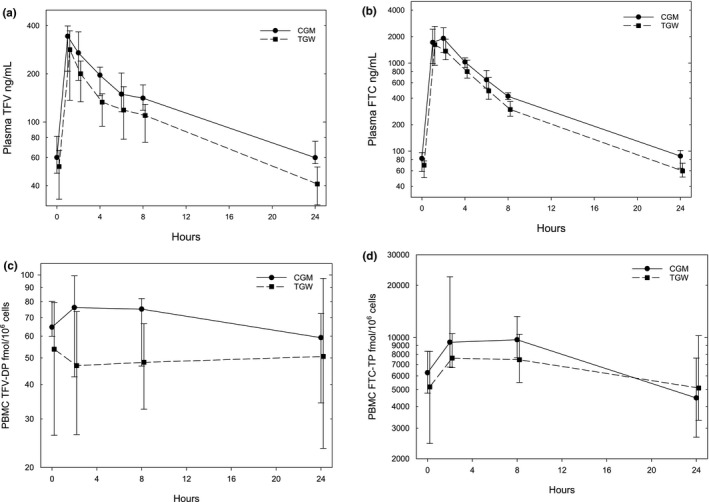
**Concentration versus time plots for plasma tenofovir (TFV, a) and emtricitabine (FTC, b), and peripheral blood mononuclear cell (PBMC) TFV diphosphate (TFV‐DP, c) and FTC triphosphate (FTC‐TP, d) comparing transgender women (TGW; dashed lines, squares) and cisgender men (CGM; solid lines, circles). Data are medians with error bars indicating lower and upper quartiles. Time values are slightly offset to avoid overlap of data.**

Concentration‐time profiles of plasma TFV and FTC (Figure 1) indicate biphasic decline after peak concentration with TGW median concentrations for both drugs falling below the concentrations for CGM at all times and progressively so after peak concentrations are achieved. PBMC TFV‐DP concentrations are nearly flat and FTC‐TP concentrations show more gently rounded peak concentrations compared to plasma FTC and demonstrated far greater variability compared to plasma concentrations.

Plasma TFV trough concentrations (C_24_) in TGW were lower by 32% (*p* = 0.010) when compared to CGM; AUC_0‐24_ trended 27% lower (*p* = 0.065) and clearance (CL_ss_/F) trended 38% higher (*p* = 0.065) in TGW compared to CGM (Table 3). Plasma TFV C_max_ and V/F were not different between gender cohorts. In TGW, plasma FTC trough (C_24_) and AUC_0‐24_ were lower by 32% (*p* = 0.038) and 24% (*p* = 0.028) respectively, while CLss/F was higher by 31% (*p* = 0.028) when compared to CGM; V/F trended higher in TGW by 26% (*p* = 0.065) when compared to CGM (Table 3). FTC C_max_ was not different between gender cohorts. There were no statistically significant PK differences between TGW and CGM in PBMC or colon tissue for any analytes.

**Table 3:** Tenofovir (TFV) and Emtricitabine (FTC) pharmacokinetic parameters

Analyte	Matrix	Units	Parameter	TGW Median (IQR)	CGM Median (IQR)	*TGW/CGM* %	*p* value^a^
TFV							
TFV	Plasma	ng/mL	C_max_	301 (210, 392)	349 (269, 398)	86	0.721
TFV	Plasma	ng/mL	C_24_	41 (30, 52)	60 (55, 76)	68	0.010
TFV	Plasma	ng‐hr/mL	AUC_0‐24_	2500 (1712, 3001)	3431 (2720, 3649)	73	0.065
TFV	Plasma	L/hr	CL_ss_/F	120 (100, 177)	87 (82, 110)	138	0.065
TFV	Plasma	L	Vz/F	2110 (1645, 3391)	2009 (1442, 2504)	105	0.574
TFV‐DP	PBMC	fmol/10^6^ cells	C_max_	69 (51, 101)	83 (67, 118)	83	0.328
TFV‐DP	PBMC	fmol/10^6^ cells	C_24_	51 (23, 97)	56 (34, 82)	90	0.982
TFV‐DP	PBMC	fmol‐hour/10^6^ cells	AUC_0‐24_	1239 (755, 1560)	1637 (1404, 1995)	76	0.121
TFV	Colon homogenate	ng/mg	C_24_	0.48 (0.17, 0.54)	0.31 (0.16, 0.83)	153	0.977
TFV‐DP	Colon homogenate	fmol/mg	C_24_	701 (368, 2694)	1329 (290, 4231)	53	0.798
TFV‐DP	Colon cells	fmol/10^6^ cells	C_24_	1238 (286, 4811)	3398 (881, 7894)	36	0.442
FTC							
FTC	Plasma	ng/mL	C_max_	2047 (1442, 2615)	2284 (1415, 2889)	90	0.645
FTC	Plasma	ng/mL	C_24_	60 (51, 73)	88 (64, 102)	68	0.038
FTC	Plasma	ng‐hr/mL	AUC_0‐24_	9682 (8512, 10,926)	12,698 (11,114, 13,683)	76	0.028
FTC	Plasma	L/hr	CL_ss_/F	20.6 (18.3, 23.6)	15.8 (14.6, 18.0)	131	0.028
FTC	Plasma	L	Vz/F	187 (173, 230)	148 (140, 162)	126	0.065
FTC‐TP	PBMC	fmol/10^6^ cells	C_max_	10,617 (7,918, 12,880)	10,943 (8702, 22,409)	97	0.382
FTC‐TP	PBMC	fmol/10^6^ cells	C_24_	5110 (3332, 10,255)	4479 (2659, 6820)	114	0.645
FTC‐TP	PBMC	fmol‐hr/10^6^ cells	AUC_0‐24_	180,869 (122,399, 203,741)	204,467 (159,963, 272,918)	88	0.281
FTC	Colon homogenate	ng/mg	C_24_	BLQ (BLQ, 0.26)^b^	BLQ (BLQ, 0.25)	–	0.706
FTC‐TP	Colon homogenate	fmol/mg	C_24_	123 (46, 183)	203 (84, 237)	60	0.279
FTC‐TP	Colon cells	fmol/10^6^ cells	C_24_	79 (BLQ^c^, 297)	180 (121, 251)	44	0.393

AUC_0‐24_, area under the concentration versus time curve from zero to 24 hours; C24, trough concentration; CGM, cisgender men; CL_ss_/F, steady‐state total clearance divided by bioavailability; C_max_, peak concentration; FTC‐TP, FTC triphosphate; IQR, interquartile range; PBMC, peripheral blood mononuclear cells; TGW, transgender women; V_z_/F, volume of distribution based on the terminal phase.

a Exact 2‐sided *p* value, Wilcoxon rank sum test, comparing transgender women (TGW) and cisgender men (CGM)

b Values below the lower limit of assay quantitation (BLQ) are included in the median (IQR) estimates; 5 CGM and 4 TGW are BLQ for FTC colon homogenates

c Among colon tissue cell FTC‐TP, 1 CGM and 3 TGW are BLQ.

### 3.3 Kinase expression

**Figure 2:**
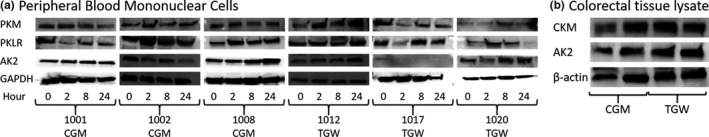
**Kinase expression analysed via immunoblotting for tenofovir‐activating kinases in peripheral blood mononuclear cells (PBMC) and colon tissue. PBMC and colon tissue were lysed and immunoblotting was performed using 50 µg of total protein lysate as described under Methods.** **(a)**, Immunoblotting of PBMC lysate for pyruvate kinase muscle (PKM), pyruvate kinase liver and red blood cell (PKLR), adenylate kinase 2 (AK2) and glyceraldehyde 3‐phosphatase dehydrogenase (GAPDH). **(b)**, Immunoblotting of colon tissue lysate for creatine kinase muscle (CKM), adenylate kinase 2 (AK2) and β‐actin. Participant identification numbers are indicated (1001, 1002, 1008, 1012, 1017 and 1020). CGM, cisgender male; TGW, transgender woman.

Expression of adenylate kinase 2 (AK2), pyruvate kinase muscle (PKM) and pyruvate kinase liver and red blood cell (PKLR) in PBMC did not differ over time at zero, two, eight and twenty‐four hours between CGM and TGW (Figure 2). Expression of creatine kinase muscle (CKM) and AK2 in colon tissue did not differ between CGM and TGW (Figure 2).

### 3.4 Pharmacodynamics

There were no significant differences in median (IQR) cumulative p24 produced by colorectal tissue explants between TGW and CGM (9.3 (8.1 to 13.6) vs. 6.8 (5.5 to 13.3) pg/mg tissue, *p* = 0.366).

### 3.5 Hormone concentrations

After seven days of TDF/FTC dosing (Day 7), concentrations for all measured hormones were unchanged compared to baseline (all *p* > 0.15) (Table 4). However, the heterogeneity in GAHT regimens and the absence of directly observed GAHT dosing meant hormone sampling before and after the TDF/FTC dosing period may not have been at steady state. After seven days of TDF/FTC dosing, oestradiol concentrations were 25‐fold higher in TGW compared to CGM. FSH, LH, total and free testosterone in CGM ranged from 2% to 8% of the concentrations measured in TGW after seven days of TDF/FTC dosing (all *p* < 0.05).

**Table 4:** Hormone Concentrations in transgender women (TGW) and cisgender men (CGM). Data other than *p* values are median (interquartile range)

Hormone	TGW Day 0^b^	TGW Day 7	CGM Day 7	Pre vs. Post TDF/FTC *p* value^a^	TGW versus CGM *p* value^a^
Oestradiol (pg/mL)	221 (60, 615)	380 (208, 437)	15 (12, 23)	0.669	<0.001
FSH (mIU/mL)	0.17 (0.10, 3.23)	0.10 (0.10, 3.87)	4.02 (2.23, 5.83)	0.806	0.047
LH (mIU/mL)	0.88 (0.13, 4.16)	0.46 (0.16, 5.63)	5.45 (2.98, 7.62)	0.626	0.048
Total Testosterone (ng/dL)	15 (10, 90)	17 (10, 297)	422 (346, 605)	0.151	0.028
Free Testosterone (ng/dL)	0.34 (0.19, 2.03)	0.35 (0.30, 3.48)	12.65 (8.10, 17.70)	0.375	0.011

a Exact 2‐sided *p* value, Wilcoxon rank sum test, comparing pre‐TDF/FTC dosing (Day 0) to post‐TDF/FTC dosing (Day 7) or comparing TGW to CGM

b All TGW had oestradiol concentrations greater than 100 pg/ML per protocol at screening. One subject fell below this value on both Day 0 and Day 8 (day of biopsy), while another fell below only on Day 0.

### 3.6 Correlation between PrEP PK parameters and hormones

Oestradiol dose and plasma FTC AUC_0‐24_ (r = −0.68, *p* = 0.007) were strongly negatively correlated. Plasma TFV trough concentrations were negatively correlated with both oestradiol dose (r = −0.53, *p* = 0.04) and Premarin dose (r = −0.54, *p* = 0.03). Correlation between GAHT doses and all other PK parameters were not statistically significant. Serum oestradiol concentrations were moderately correlated with plasma C_24_ concentrations of TFV (r = −0.54, *p* = 0.03) and FTC (r = −0.52, *p* = 0.04). FSH measurements were moderately correlated with plasma C_24_ FTC concentrations (r = 0.61, *p* = 0.02) and PBMC TFV‐DP C_max_ (r = 0.58, *p* = 0.02). Other hormone and TDF/FTC PK parameters were not statistically significantly correlated.

### 3.7 Renal function

Renal function in TGW was significantly higher when compared to CGM using all three measures—creatinine clearance (CrCl) estimated with the Cockcroft‐Gault equation and glomerular filtration rate (GFR) estimated using both MDRD and CKD‐EPI equations (all *p* < 0.01) (Table 5). When we substituted the female term for the male term in the two GFR estimating equations for the TGW on GAHT, the differences between TGW and CGM largely disappeared and were no longer statistically different. Estimated CrCl remains significantly higher for TGW compared to CGM when substituting female for male for TGW. Plasma TFV and FTC trough concentrations (C_24_) and exposure (AUC_0‐24_) were all strongly negatively correlated with all three measures of renal function (r range −0.65 to −0.82, *p* ≤ 0.02 in all comparisons). When the formulas for females were substituted for TGW, the correlation dropped slightly.

**Table 5:** Estimated creatinine clearance (CrCl) and estimated glomerular filtration rate (eGFR) on study day 7

Physiologic variables	TGW Median (IQR^a^)	CGM Median (IQR)	TGW/CGM, %	*p* value^b^
Serum Creatinine (mg/dL)	0.80 (0.80, 0.88)	1.05 (1.00, 1.18)	76	0.002
CrCl Cockcroft‐Gault (mL/min)	204 (120, 218)	108 (83, 125)	188	0.008
eGFR MDRD (mL/min/1.73 m^2^)	130 (117, 142)	89 (79, 98)	146	0.002
eGFR CKD‐EPI (mL/min/1.73 m^2^)	133 (124, 142)	114 (109, 118)	116	0.003
CrCl Cockcroft‐Gault [TGW = F] (mL/min)^c^	174 (102, 185)	109 (83, 125)	160	0.035
eGFR MDRD [TGW = F] (mL/min/1.73 m^2^)^c^	97 (86, 105)	89 (79, 98)	109	0.442
eGFR CKD‐EPI [TGW = F] (mL/min/1.73 m^2^)^c^	107 (100, 118)	98 (81, 106)	109	0.315

CGM, cisgender men; CKDEPI, Chronic Kidney Disease Epidemiology Collaboration equation; IQR, interquartile range (lower quartile, upper quartile); MDRD, Modification of Diet in Renal Disease Study equation; TGW, transgender women.

a Interquartile range

b Exact 2‐sided *p* value, Wilcoxon rank sum test, comparing TGW and CGM

c CrCL and eGFR estimated using the female term replacing the male term in the equation for TGW.

### 3.8 Histology

Surface denudation, lamina propria haemorrhage and apoptotic bodies scores did not differ between TGW and CGM.

## 4 Discussion

We found a 24% to 32% reduction in plasma TFV and FTC AUC_0‐24_ and C_24_ in TGW on a variety of oestrogen regimens when compared to CGM. The iFACT study also showed a reduction in plasma TFV—18% C_24_ and 12% AUC_0‐24_—in 20 TGW on a standardized oestrogen/cyproterone regimen 20. The different magnitude of TFV reductions may have been due to study differences, for example, higher oestrogen doses in some of our participants, different anti‐androgens, and use of parallel group design (with trending higher BMI) in our study. Another PrEP‐GAHT interaction study found a sevenfold reduction in rectal tissue homogenate TFV‐DP:dATP ratio in TGW on GAHT, also raising concern regarding PrEP efficacy 21. In this regard, our active phosphorylated TFV and FTC analytes were not statistically significant. However, our assessment of these intracellular readouts is limited by greater assay variability, small sample size and sparse sampling. In addition, detailed examination of the plasma ARV and hormone concentrations indicate GAHT non‐adherence in some participants possibly related to a drug‐drug interaction not at steady‐state, which would result in a delayed impact on the relatively longer half‐life of the phosphorylated drug analytes when compared to parent drug in plasma.

The magnitude of reduced plasma TFV and FTC exposure associated with GAHT regimens in these studies is consistent with taking only five doses (Hopkins) or six doses (iFACT) per week when compared to taking 7 daily TDF/FTC doses each week, based on HPTN 066 directly observed TDF/FTC dosing 12. In HPTN 066, which reported combined results for CGM and cisgender women not on GAHT, the median (IQR) steady‐state TFV and FTC serum concentrations were 52 ng/mL (49 to 56) and 71 ng/mL (68 to 82) respectively 12. The iPrEx study demonstrated highly effective oral TDF/FTC as PrEP with only four doses per week, motivating the Ipergay and Prevenir on demand 2‐1‐1 TDF/FTC regimens, which demonstrated very high 86% and 100% HIV protection respectively, in MSM and TGW 22, 23, 24. However, there were too few TGW in those studies to draw conclusions regarding PrEP effectiveness on GAHT. Theoretically, one would expect the combination of reduced concentrations from both the 2‐1‐1 regimen and GAHT to result in a dose frequency equivalent of two to three doses per week. At this dose frequency, PrEP efficacy in the iPrEx models would predict reductions in PrEP efficacy 22. Accordingly, it seems prudent to recommend against the 2‐1‐1 regimen in TGW on GAHT, at least until more rigorous PK studies address optimal PrEP dosing in TGW.

The mechanism behind lower plasma TFV and FTC in the setting of exogenous oestrogen (and many also with spironolactone) is not clear. The TFV plasma clearance (CLss/F) trends higher in TGW without parallel difference in volume of distribution (Vz/F). Because clearance and volume are influenced by oral bioavailability in when dosing is oral, the difference in clearance and volume changes suggests bioavailability was not a major factor. For FTC, both clearance and volume changed similarly (with small differences in magnitude and statistical significance). Therefore, bioavailability or volume may between cohorts. However, we have no explanation for these and find more consistent evidence for a difference in clearance. TGW had markedly higher creatinine clearance and eGFR when compared to CGM participants, which might account for the major mechanism for reduced concentrations of TFV and FTC, both of which are primarily eliminated through renal clearance. We also observed a correlation of serum oestrogen concentration with both the TFV and FTC reduction and the renal glomerular functional increase. We found that oestradiol does not affect the kinases that phosphorylate TFV in plasma and colon tissue, but this might have been the result of off‐setting decreases in plasma parent drug PK and increases in kinase effects (predicted by in vitro data) 9.

Whether this difference in renal function is due to oestradiol or simply an association is unclear. Prior studies have shown both increasing renal function with acute use of oestrogens and decreasing renal function with chronic oestrogen dosing 25, 26. A more mechanistic explanation may involve the oestrogen effect in upregulation of angiotensin‐2 receptor (AT_2_R) expression resulting in vasodilation and increased clearances shown in rodents, though supraphysiologic oestrogen concentrations result in vasoconstriction 27, 28, 29, 30, 31. In addition, testosterone downregulates AT_2_R expression, resulting in decreased vasodilation 32. Since testosterone concentrations are reduced by a variety of drugs used in GAHT regimens, resulting in greater than 20‐fold lower free testosterone in our TGW compared to CGM participants, this may be an additional mechanism of enhanced renal clearance of TFV and FTC. We did not assess angiotensin receptor expression to explore this potential explanation.

While we did not identify an impact of PrEP drug on oestrogen concentrations, the heterogeneity of oestrogen formulations and doses among the GAHT prevented us from sampling plasma to assess oestrogen concentrations reliably at times coinciding with pseudo steady‐state conditions. In addition, we did not use directly observed dosing of all GAHT drugs (unlike with TDF/FTC dosing). As a result, we are not highly confident about the finding of no TDF/FTC impact on oestrogen concentrations. In contrast, the iFACT study team was able to employ a standardized GAHT regimen of oestradiol and cyproterone acetate and demonstrated that TDF/FTC dosing did not affect oestrogen concentration. Furthermore, since GAHT regimens are often adjusted to achieve desired subjective and objective effects in clinical practice, even modest TDF/FTC impacts on oestrogen concentration would be corrected by usual clinical practice.

Our study also suggests caution when using pharmacologically based adherence benchmarks established in one population when applied to another. In iPrEx, some of the concentration difference noted in TGW on GAHT may be attributable to GAHT‐PrEP interactions in addition to adherence. A clearer example of adherence mischaracterization would be in pregnant women in whom plasma TFV concentrations are 58% lower than in non‐pregnant women when controlling for adherence 33.

## 5 Conclusions

We demonstrated that GAHT regimens taken by TGW in our study resulted in greater than 20% reduction in both TFV and FTC taken as oral daily PrEP, possibly because of increases in renal clearance. The iFACT study made similar findings. We caution that the combination of a GAHT regimen and the on demand 2‐1‐1 PrEP regimen may result in TFV and FTC concentrations that fall below those associated with high levels of HIV protection. Conversely, neither our study nor the iFACT study demonstrated any effect of daily TDF/FTC on oestrogen concentrations providing needed reassurance for TGW concerned that PrEP may negatively affect their GAHT regimen. More rigorous study designs are essential to explore the mechanism of the oestrogen effect, better define the magnitude of related active TFV and FTC concentration changes in colorectal tissue, and confirm our dosing cautions with regard to GAHT and on demand PrEP regimens.

## Competing interests

Craig Hendrix, Edward Fuchs, Jennifer Breakey, Mark Marzinke and Rahul Bakshi receive research support from Gilead Sciences through a contract with and managed by Johns Hopkins University School of Medicine. The other authors note no conflicts of interest.

## Authors’ contributions

ES, MAM, EJF, AH, RB, WA, JB, TP, TB, NNB and CWH performed the research. ES, MAM, EJF, AH, JB, NNB and CWH designed the research study. ES and CWH analysed the data and drafted the manuscript. All authors read successive versions of the manuscript and approved the final manuscript.
